# Low frequency ultrasonic pulse-echo datasets for object detection and thickness measurement in concrete specimens as testing tasks in civil engineering

**DOI:** 10.1016/j.dib.2023.109233

**Published:** 2023-05-12

**Authors:** Stefan Maack, Stefan Küttenbaum, Benjamin Bühling, Kerstin Borchardt-Giers, Norman Aßmann, Ernst Niederleithinger

**Affiliations:** Bundesanstalt für Materialforschung und Prüfung (BAM), Unter den Eichen 87, Berlin 12205, Germany

**Keywords:** Pulse-echo method, Non-destructive testing, Reconstruction algorithm, SAFT, Evaluation, Validation, Piezoelectric transducer, Civil engineering

## Abstract

The dataset contains raw data gathered with the ultrasonic pulse-echo method on concrete specimens. The surfaces of the measuring objects were automatically scanned point by point. Pulse-echo measurements were performed at each of these measuring points. The test specimens represent two typical testing tasks in construction industry: the detection of objects and the determination of dimensions to describe the geometry of components. By automating the measurement process, the different test scenarios are examined with a high repeatability, precision and measuring point density. Longitudinal and transversal waves were used and the geometrical aperture of the testing system was varied. The low-frequency probes operate in a range of up to approximately 150 kHz. In addition to the specification of the geometrical dimensions of the individual probes, the directivity pattern and the sound field characteristics are provided. The raw data are stored in a universally readable format. The length of each time signal (A-scan) is two milliseconds and the sampling rate is two mega-samples per second. The provided data can be used for comparative studies in signal analysis, imaging and interpretation as well as for evaluation purposes in different, practically relevant testing scenarios.

**Specifications Table**

Every section of this table is mandatory. Please enter information in the right-hand column and remove all the instructions in gray, italicised text.

**Table:** 

Subject	Engineering
Specific subject area	Automated ultrasonic echo measurements with varying testing scenarios on concrete specimens for civil engineering applications.
Type of data	Time series
How the data were acquired	Automated scanning system: In-house development by BAM Probes: M2502 – Dual Aperture Shear Wave DPC Transducer Array (Acoustic Control Systems - ASC Group), M2503 – Dual Aperture Longitudinal Wave DPC Transducer Array (Acoustic Control Systems - ASC Group) Driving signal generator: In-house development by BAM Data Acquisition: USB-6361 (National Instruments Corp.) Software: LabVIEW© (National Instruments Corp.- Scanner control and data acquisition developed by BAM, Germany)
Data format	Raw data
Description of data collection	The measuring data was recorded on two concrete objects with an automated scanner system, which allows the sampling of dense measuring point grids ensuring a high positioning accuracy. The test specimen were manufactured using the same concrete mixtures and external geometrical dimensions. The objects differ only with regard to the installation of structural components, i.e., tendon ducts.
Data source location	• Institution: Bundesanstalt für Materialforschung und -prüfung (BAM) • City/Town/Region: Berlin • Country: Germany
Data accessibility	Repository name: Harvard Dataverse Data identification number: 10.7910/DVN/9EID5D Direct URL to data: https://doi.org/10.7910/DVN/9EID5D Data identification number: 10.7910/DVN/NUU0WZ Direct URL to data: https://doi.org/10.7910/DVN/NUU0WZ
Related research article	S. Maack, S. Küttenbaum, E. Niederleithinger, Practical procedure for the precise measurement of geometrical tendon positions in concrete with ultrasonic echo. MATEC Web Conf., 364 (7–8) (2022), 3008. https://doi.org/10.1051/matecconf/202236403008

## Value of the Data


 

• The challenge in testing concrete with the ultrasonic echo method is that on-site boundary conditions, e.g., on construction sites, may differ significantly when examining different components in practice. It is therefore important to evaluate and further develop the performance of evaluation algorithms used for ultrasonic data interpretation. The provided data cover an important area of common testing tasks.
• Both investigated specimens have the same external geometry and were made from the same concrete mixture. Differences exist about the mounting of typical construction elements (in this particular case: tendon ducts). The data can thus be used to evaluate the effects of different evaluation and analysis methods for various testing scenarios and to validate newly developed methods.
• Users of the ultrasonic technique who intend to evaluate their evaluation methods, hardware and software developers, as well as researchers working in the fields of data processing and reconstruction methods for pulse-echo methods will benefit in particular.
• The dataset is suitable for the training and qualification of personnel working with non-destructive testing methods.
• The data sets represent different testing scenarios and are based on different wave types. Thus, the performance of evaluation approaches can be compared (e.g., in the scope of interlaboratory tests). Furthermore, as the measuring objects differ only in one feature (tendon ducts mounted / no tendon ducts installed), it can be assessed to what extent the construction elements affect spatial wave propagation.
• The data set is also suitable to develop and evaluate new designs of experiments based on the investigated test specimens.

## 1 Objective

The provided ultrasonic data [1,2] were recorded on two concrete specimens of the Federal Institute of Materials Research and Testing (BAM) in Berlin, Germany. The internal specimen designations are “Pk050” and “Pk226” (specimen in German: Probekörper (Pk)) [3]. The test specimens are made of the same concrete mixture and differ essentially by a single feature which is related to their internal structure: Pk050 does not include tendon ducts, while four tendon ducts were mounted during construction of Pk226. The measurements were conducted using the pulse-echo method. The transmitting and receiving transducers are placed on the same surface of the measuring object. The upper surfaces of the respective specimens were defined as measuring areas (X-Y-plane). The objective in the design and manufacturing process of the specimens was to investigate the possibilities of determining geometrical dimensions (e.g., component thicknesses) and geometrical positions of internal components (e.g., prestressing steel) using non-destructive testing methods.

By publishing the datasets, the method for reconstruction of ultrasonic measurement data presented in the related publication [4] can be evaluated directly. Furthermore, the publication results in the possibility to improve the reconstruction results described in [4] by developing or applying other methods.

## 2 Data Description

The data are raw data recorded point by point in a defined measuring grid on the surface of the two specimens. At each measuring point, a pulse-echo measurement of the excited sound pulse is performed. The time signal resulting from one pulse-echo measurement is usually referred to as an A-scan in ultrasonic testing [5]. Such an A-scan maps the sound pressure of the received signal as amplitude in relation to a certain time of flight of the pulse. The sampling rate is f = 2 MHz. This corresponds to a time increment between two sampling values of Δt = 0.5 µs. The length of one A-scan is t = 2000 µs, so that one time signal consists of 4000 samples. The stored data are 16-bit integers (signed) in the range of A = ±2000 mV.

**Fig. 1:**
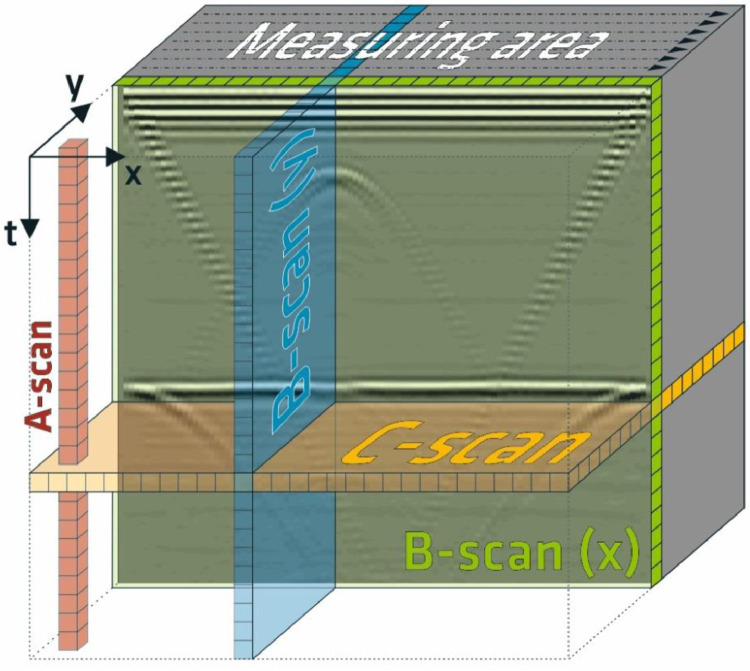
Overview of the designations of the different viewing planes which can be derived from point (A-scan) and line (B-scans) as well as area measurements (C-scan); extracted from [7], translated).

By measuring the A-scans at various points within a defined area, a 3D-dataset containing volume information can be compiled (cf. Fig. 1). The data are provided in different data formats and types. On the one hand, individual B-scans (a B-scan consists of a number of A-scans sampled next to each other on a measuring line, see Fig. 1) are made available in the generally readable CSV format (*.csv-files) [6]. On the other hand, the measuring data are provided in a NumPy format (*.npy-files, Python versions 3.8) as coherent 3D-datasets.

**Fig. 2:**
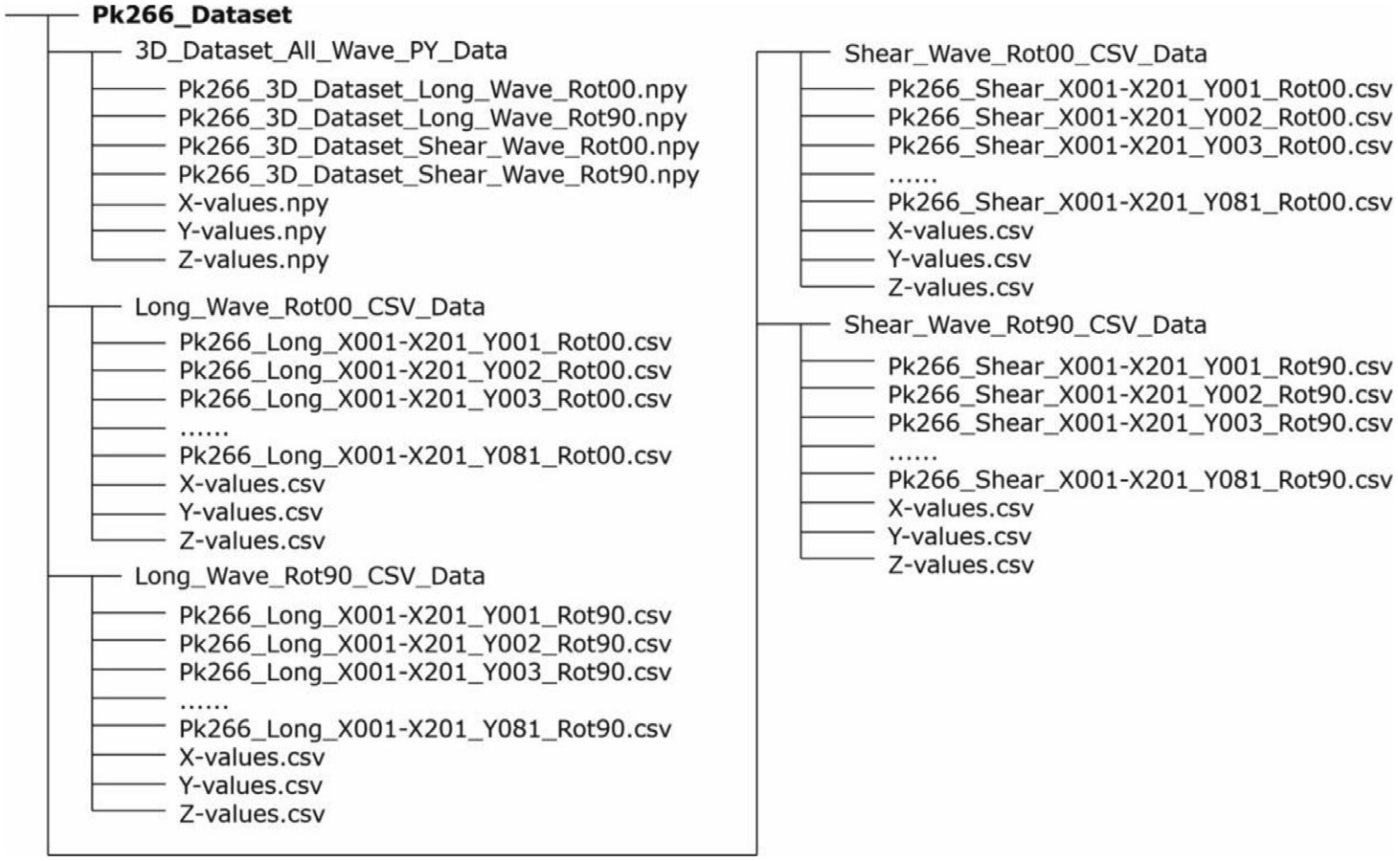
Data tree including the subfolder structure of specimen Pk266 and overview of the files stored in different formats (in excerpts). The dataset of specimen Pk050 has an identical structure.

Fig. 2 indicates the logical data structure of the different file formats and data storage types.

**Fig. 3:**
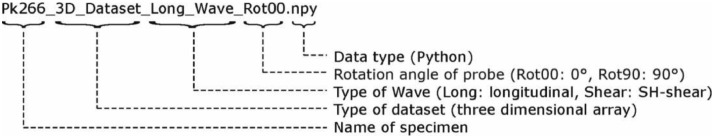
Exemplary description of the file names stored in NumPy-format (*.npy).

The subfolder ”3D_Dataset_All_Wave_PY_Data“ contains the 3D-datasets in NumPy format (*.npy-files). The four data sets differ regarding the respective wave type (longitudinal, transversal (shear)) and orientation of the probe (Fig. 17, Fig. 18) on the concrete surface determining the direction of polarization. Three additionally provided vectors comprise the geometrical orientation of the measuring points in X- and Y-direction in millimeters [mm] as well as the time resolution in Z-direction in microseconds [µs]. The designation for the 3D datasets is given in Fig. 3.

**Fig. 4:**
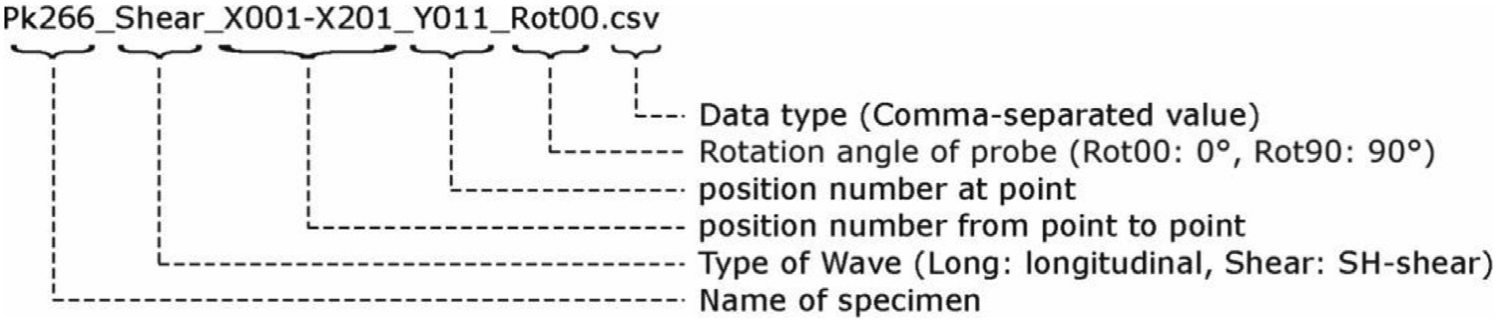
Exemplary description of the file names stored in CSV-format (*.csv).

The data tree (Fig. 2) contains four further subfolders in which the 2D datasets are stored in CSV format (*.csv-files). The folder names indicate the wave type (longitudinal, transversal (shear)) and the probe orientation (e.g., Rot00 corresponds to a 0° rotation). In contrast to the Python datasets, the CSV datasets are stored in individual B-scans along the X-axis. In addition, each folder stores three vectors, again describing the geometrical measuring point information (X- and Y-direction in millimeters [mm]) and the temporal resolution (Z-direction in microseconds [µs]). The designation for the 2D datasets is given in Fig. 4.

**Fig. 5:**
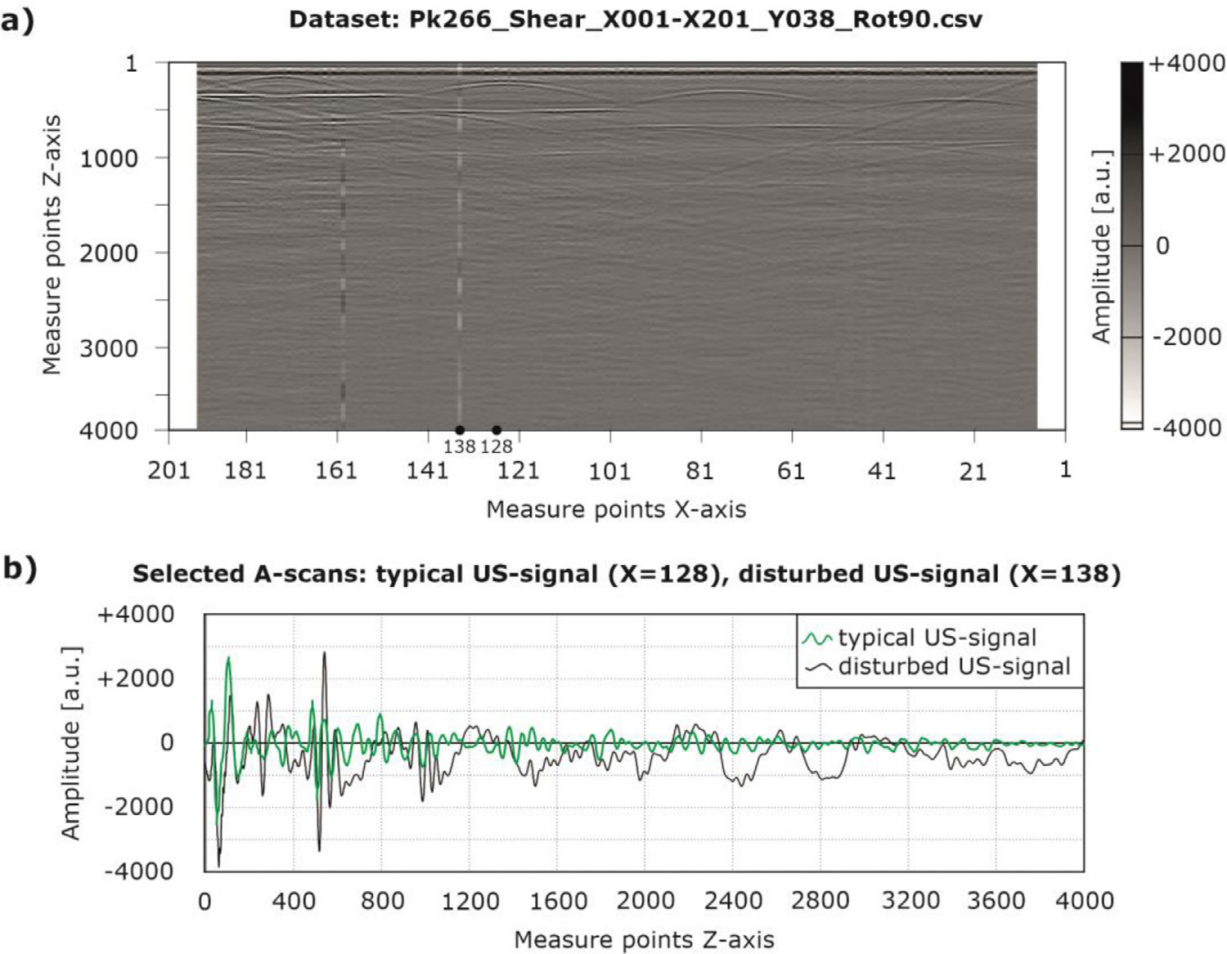
a) Imaging of a B-scan generated from the CSV dataset “Pk266_Shear_X001-X201_Y038_Rot90.csv” and b) typical and disturbed US-signals (A-scans) at the positions X = 128 and X = 138.

In the upper part of Fig. 5, a) a B-scan color-coded in grayscale and generated from the CSV dataset “Pk266_Shear_x001-x201_y038_Rot90.csv” is shown. In the lower part of Fig. 5, b), two A-scans are visualized at the positions X = 128 (green line) and X = 138 (black line). The A-scan at position X = 138 is overlaid by an interfering signal. This effect is caused by the frame of the scanner system (see chapter ”Experimental design, materials and methods“) and can be eliminated using digital filters. Additionally, it should be taken into account, that the first (X = 1 to X = 7) and last (X = 195 to X = 201) A-scans used to derive a B-scan do not contain measuring data (Fig. 5, a)). In these areas, the probe could not be completely mounted on the component surface (individual transducers were located outside the measuring area). However, in order to represent the actual geometrical dimensions of the specimens, respective zero vectors are provided.

## 3 Experimental Design, Materials and Methods

### 3.1 Design of Experiment

**Fig. 6:**
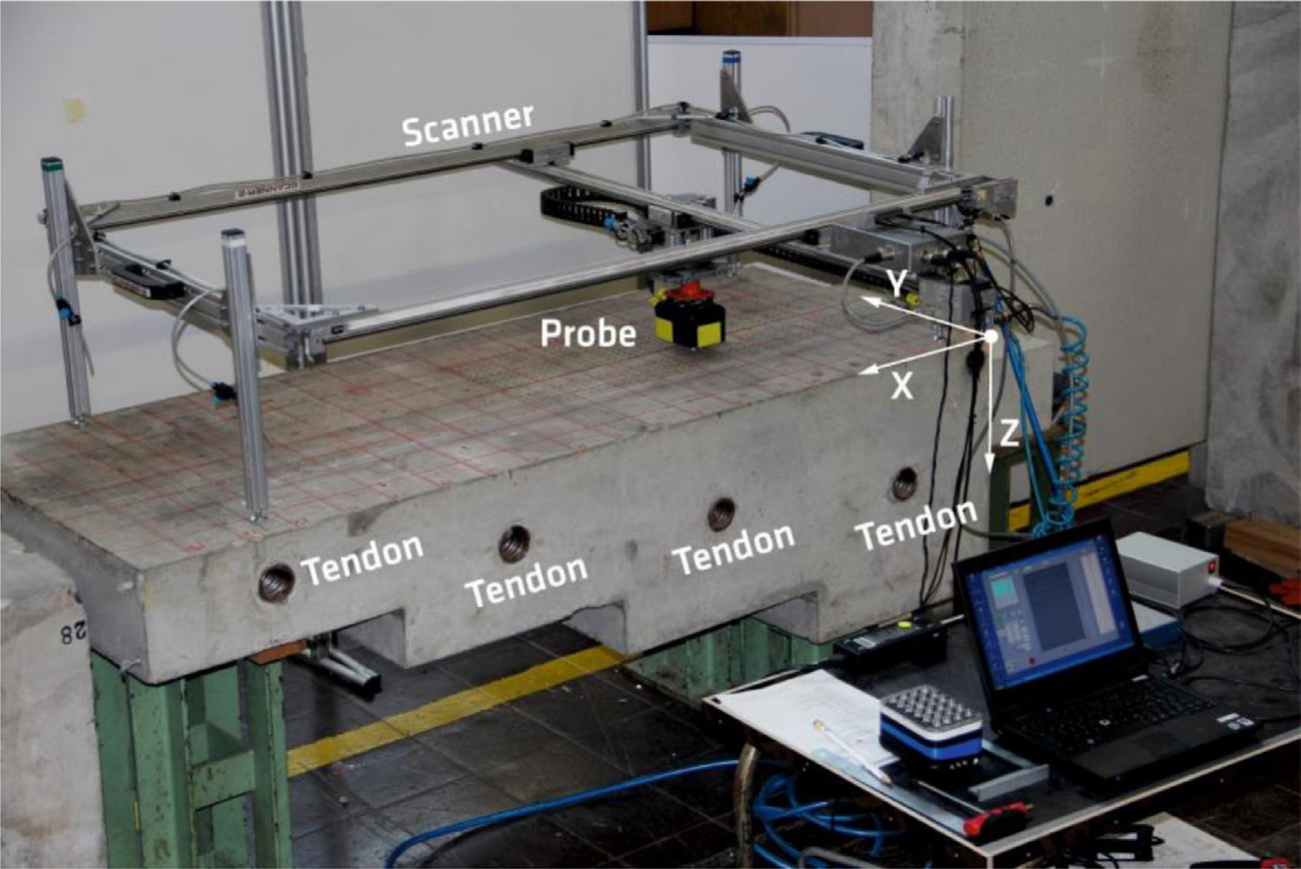
Experimental set-up for automated data acquisition on BAM-test specimen Pk266.

At BAM, two test specimens (Pk050 and Pk266) were manufactured for the evaluation and verification of the performance of non-destructive testing methods. They represent different scenarios relevant in practice. Both specimens have the same geometrical dimensions. The difference is that four tendon ducts are installed in Pk266. Fig. 6 shows the experimental setup for recording the ultrasonic measurement data on the Pk266 specimen.

**Fig. 7:**
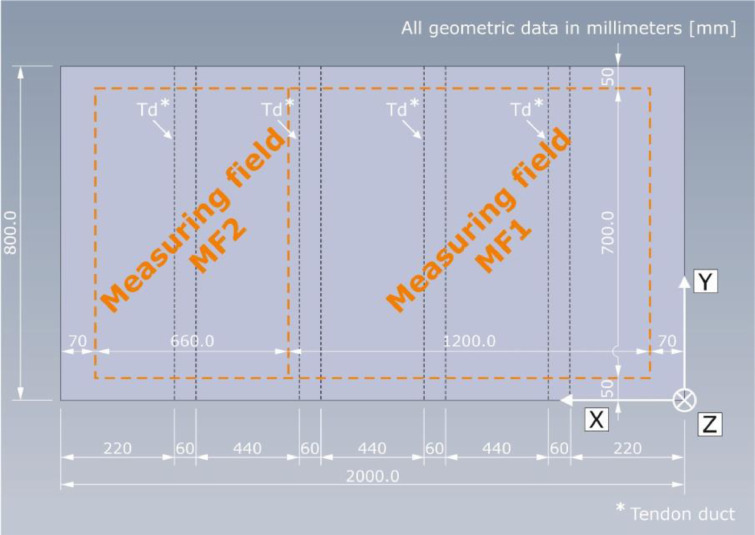
Geometrical information on the two measuring fields MF1 and MF2 as a top view of specimen Pk266.

The automated scanner system developed at BAM moves the probe from on measuring point to the next and scans the component surface in a measuring grid. The measuring point spacing in X- and Y-direction is a_X_ = a_Y_ = a = 10 mm. As the length of the specimens Pk050 and Pk266 in X-direction is larger than the range of the scanning system, each of their upper surfaces has been divided into two measuring fields (Fig. 7). Measuring field 1 (MF1) has a length of X_MF1_ = 1200 mm; measuring field 2 (MF2) a length of X_MF2_ = 660 mm. These were then digitally merged into one measuring field and stored.

To be able to mount the whole probe on the measuring surface, a distance to the component edges of X = 70 mm in X-direction and Y = 50 mm in Y-direction needs to be maintained. The resulting measuring area is visualized by the orange dashed line in Fig. 7. To match the component surface dimensions and the measuring grid coordinates, the data gathered outside the aforementioned area was replaced by zero vectors (see chapter “Data description”).

The essential information about the data acquisition is provided in Table 1 for the four different datasets.

**Table 1:** Information about the data acquisition.

Dataset	Probe type	Measuring field dimension (X × Y)	Grid space	Samples per Scan	Sampling frequency	Amplitude range
*Long*Rot00*	M2503	1860 mm × 700 mm	10 mm	4000	2 MHz	±500 mV
*Long*Rot90*	M2503	1860 mm × 700 mm	10 mm	4000	2 MHz	±500 mV
*Shear*Rot00*	M2502	1860 mm × 700 mm	10 mm	4000	2 MHz	±2000 mV
*Shear*Rot90*	M2502	1860 mm × 700 mm	10 mm	4000	2 MHz	±2000 mV

All data are 16-bit (signed) integer values.

### 3.2 General Information About Both Specimens

Both investigated test specimens were manufactured as part of the research project ”FOR 384: Zerstörungsfreie Strukturbestimmung von Betonbauteilen mit akustischen und elektromagnetischen Echo-Verfahren“, which received funding from the Deutsche Forschungsgemeinschaft (DFG) in the years 2000 to 2007 (grant number: 5466784) [3].

**Fig. 8:**
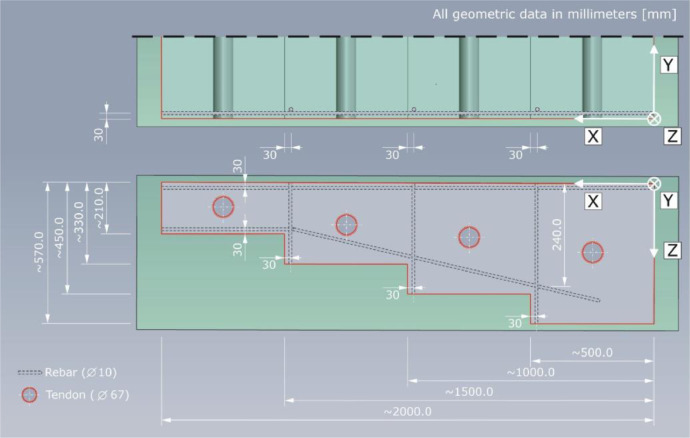
Schematic structure of the test specimens according to the basic design principles of FOR 384 [3]; here: Pk266.

**Fig. 9:**
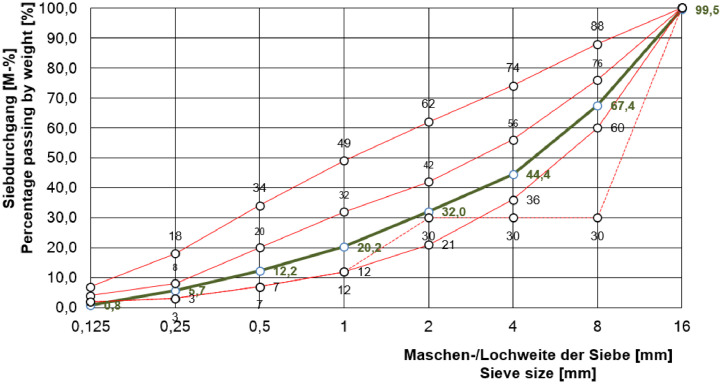
Recalculation of the grading curve A/B 16 (green solid line).

The main idea was to design specimens with equal (outer) geometrical dimensions and material properties. The stepped components comprise of four areas with a length of about 500 mm in X-direction, within which the component thickness is considered constant. To avoid concrete cracking, near-surface steel bars reinforce the objects, as shown in Fig. 8, in a mounting depth of a_X,Y,_*_Z_* = 30 mm. The steel bars have a diameter of D = 10 mm and are made of steel grade St 37 (German Industrial Standard DIN 17,100). The concrete mixture (water-cement ratio, cement type (CEM I 32.5 R), etc. are summarized in Table 2) and the grading curves are identical. The maximum aggregate diameter is D = 16 mm. The grading curve shown in Fig. 9 is recalculated (original protocol are no available) based on the known type of aggregate and k-value (k = 4.19, sum of the residues in the sieves indicated in vol.%.).

**Table 2:** Material properties of specimen concrete based on German Industrial Standard DIN 1045 (adopted from BAM protocol).

Original description (german)	Translated description (english)	Specific value
Wasserzementwert (w/z)	Water-cement ratio (w/c)	0.47
Wasser (w)	Water (w)	193.0 kg
Zement (z)	Cement (c)	411.0 kg
Konsistenz	Consistency	KR
Luftporengehalt	Air void content	1%
Zuschlag (gesamt)	Concrete aggregate (total)	1762 kg
Sieblinie	Grading curve	A/B 16
Zementart und Festigkeitsklasse	Cement type and strength class	CEM I 32.5 R
Gesteinsart: Kiessand, Oder-Material (Fa. Quarzwerke Ottendorf-Okrilla)	Type of aggregate: gravel sand (Com. Quarzwerke Ottendorf-Okrilla)	—
Rohdichte Festbeton	Bulk density hardened concrete	2383 kg/m³
Druckfestigkeit (f_ck,cube_)	Compressive strength (f_ck,cube_)	48.7 N/mm²

The geometrical dimensions and the individual specimen features are explained below. The lateral reinforcement will be delimited due to its minor effects on the measuring data.

### 3.3 Specimen Pk050

**Fig. 10:**
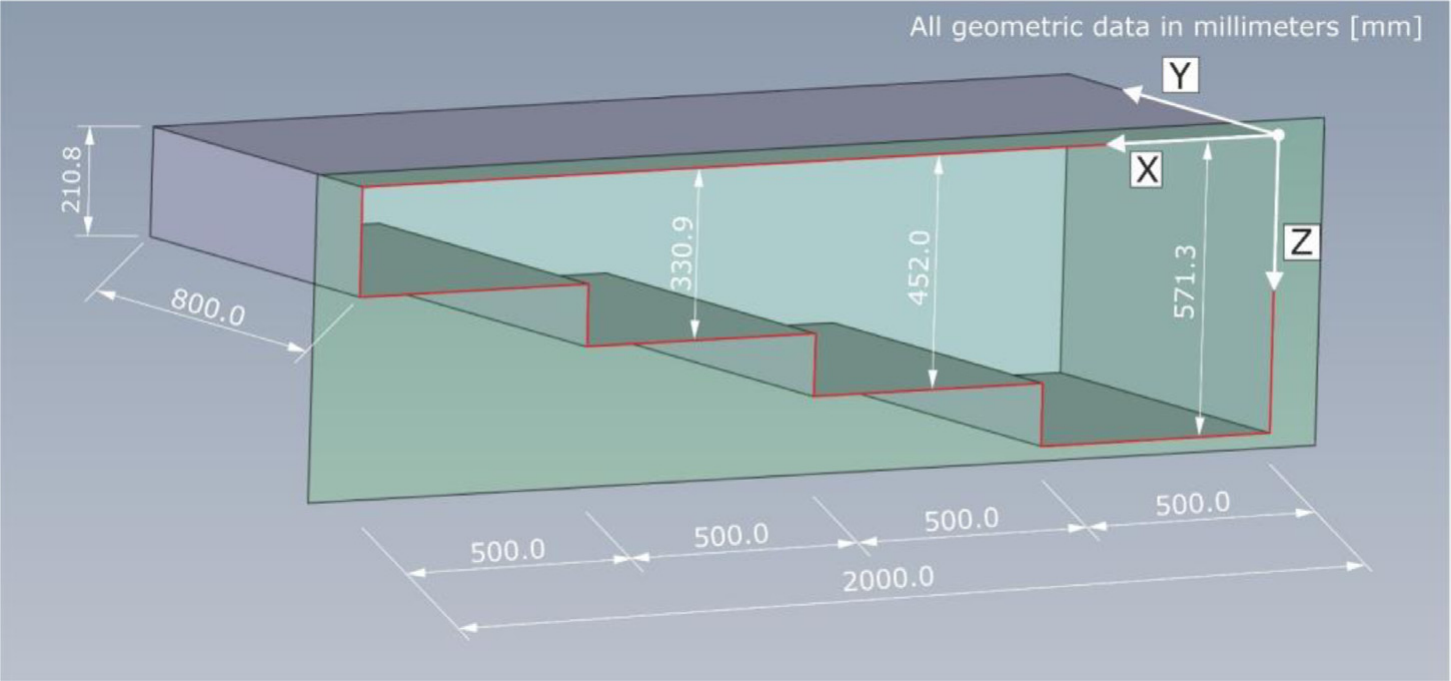
3D view of test specimen Pk050.

The 3D view in Fig. 10 visualizes the geometrical dimensions of test specimen PK050 which does not contain any tendon ducts. The indicated thicknesses of the single component steps are mean values corresponding to the average of the thicknesses measured at each respective step corner. All surfaces have a production-related imperfection of about ±1 mm.

The dimensions provided in Fig. 10 refer to the mean thicknesses of the respective steps, while Z = 0 mm corresponds to the measuring area, namely the upper component surface. The standard deviations attributed to the thickness measurements are provided in Table 3.

**Table 3:** Geometrical dimensions of test specimen Pk050 with indication of the standard deviation.

Geometric dimension	X-direction	Y-direction	Z-direction
Length / Width	2000.0 mm	800.0 mm	4 steps
Depth (step 1)	0.0–500.0 mm	800.0 mm	571.3 ± 0.69 mm
Depth (step 2)	500.0–1000.0 mm	800.0 mm	425.0 ± 1.03 mm
Depth (step 3)	1000.0–1500.0 mm	800.0 mm	330.9 ± 0.69 mm
Depth (step 4)	1500.0–2000.0 mm	800.0 mm	210.8 ± 0.57 mm

**Fig. 11:**
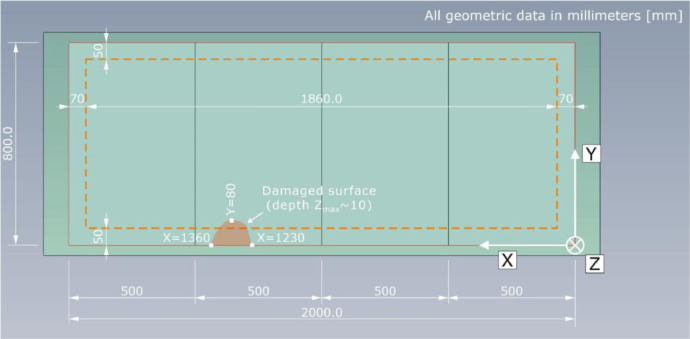
Technical drawing of test specimen Pk050; top view with indication of the spalling on the concrete surface.

The top view on specimen Pk050 is given in Fig. 11. The indicated damaged surface in the area between X = 1230 mm and X = 1360 mm as well as Y = 0 mm and Y = 80 mm, in which concrete spalling with a maximum depth of about Z = 10 mm occurred, should be considered as this influence the recorded signals. The dashed orange line, again, shows the boundaries of the measuring field.

Table 3 summarizes the geometrical dimensions of specimen Pk050 based on the chosen coordinate system. The specified thicknesses of the individual component steps include the empirical standard deviation of the thickness measurements in the respective four step corners.

### 3.4 Specimen Pk266

**Fig. 12:**
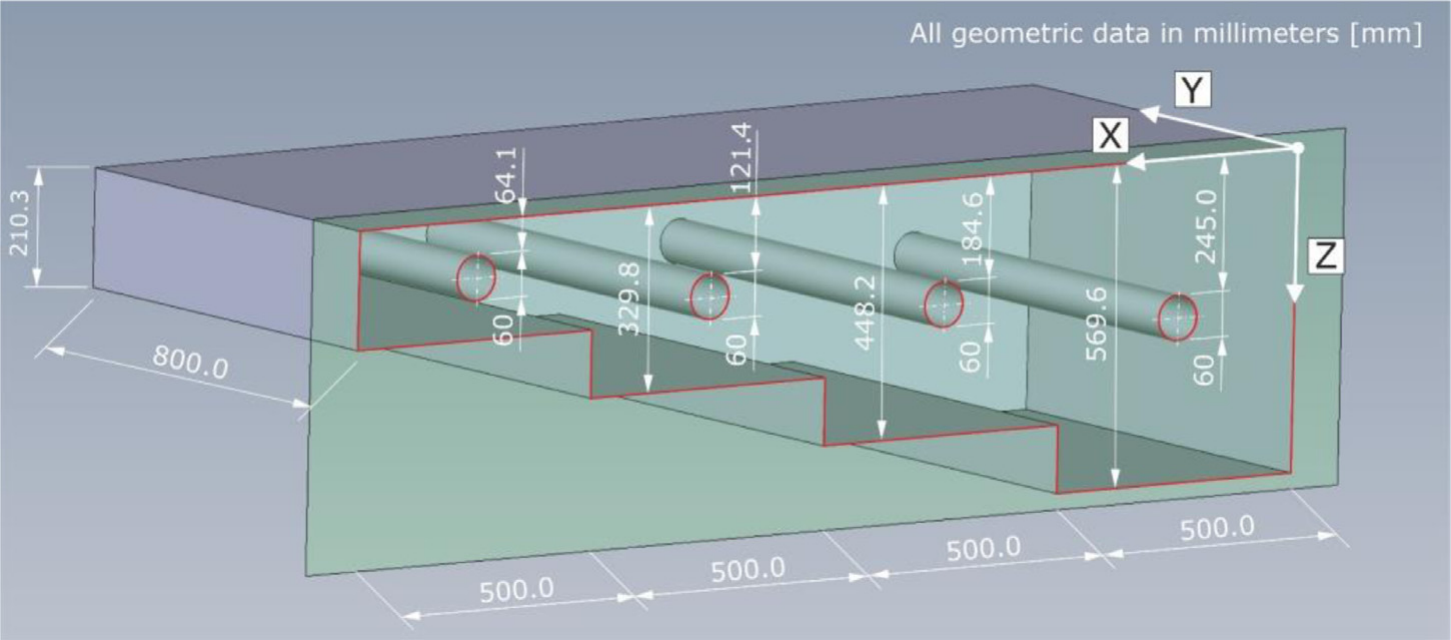
3D view of test specimen Pk266. The depth positions of the tendon ducts refer to the measuring area (Z = 0 mm) taking into account the inner duct diameter D_i_ = 60 mm.

The 3D view of specimen Pk266 can be found in Fig. 12, including the geometrical dimensions and depth positions as well as diameters of the four tendon ducts. The component thicknesses are mean values calculated from twelve measurements performed at the front sides of each step. The specified mounting depths of the tendon ducts are the mean of one measurement on each of both front sides of the specimen and describe the spacing between measuring area (Z = 0 mm) and the facing duct surface (i.e., the concrete cover). The outer diameter of the tendon ducts is D_o_ = 67 mm; the diameter D_i_ = 60 mm (see also Fig. 14). The concrete surface imperfection is about ±1 mm.

**Fig. 13:**
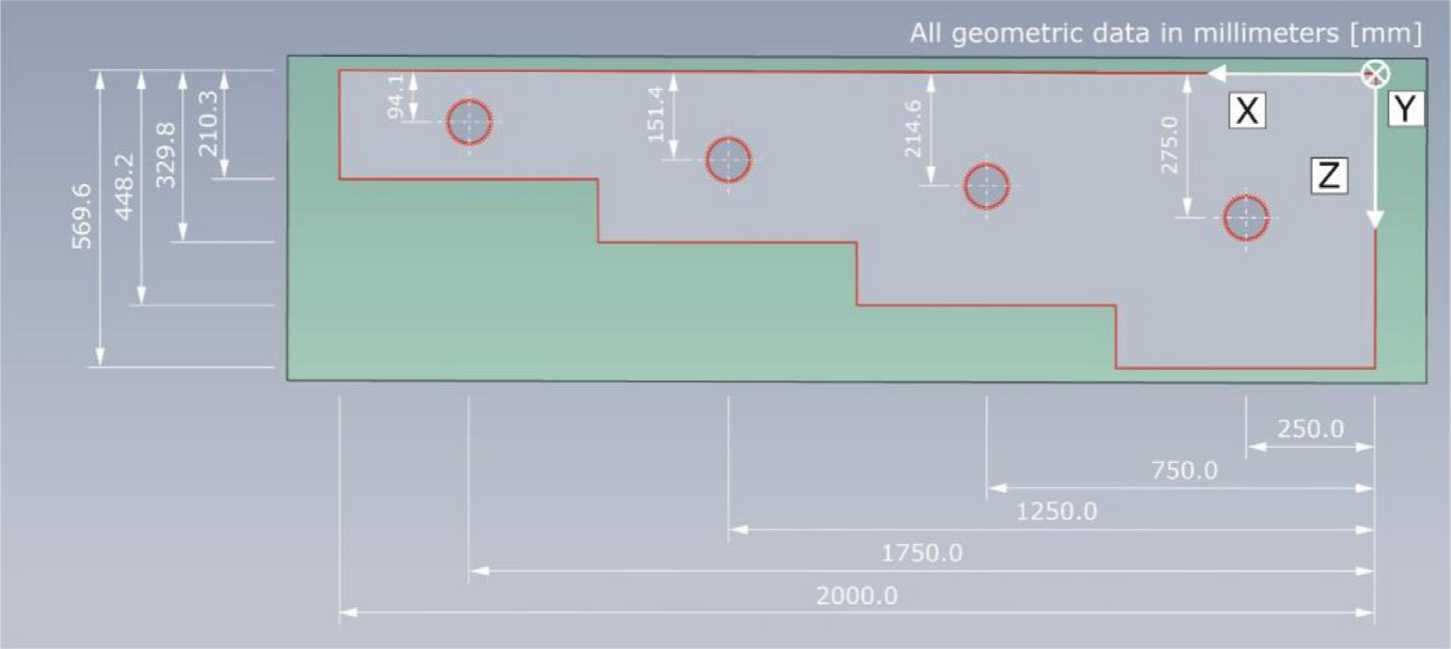
Lateral view of test specimen Pk266.

The lateral view in Fig. 13 shows one sectional plane of specimen Pk266. In addition to the thicknesses of the four component steps, the positions of the tendon ducts are indicated in relation to their symmetry axis.

**Fig. 14:**
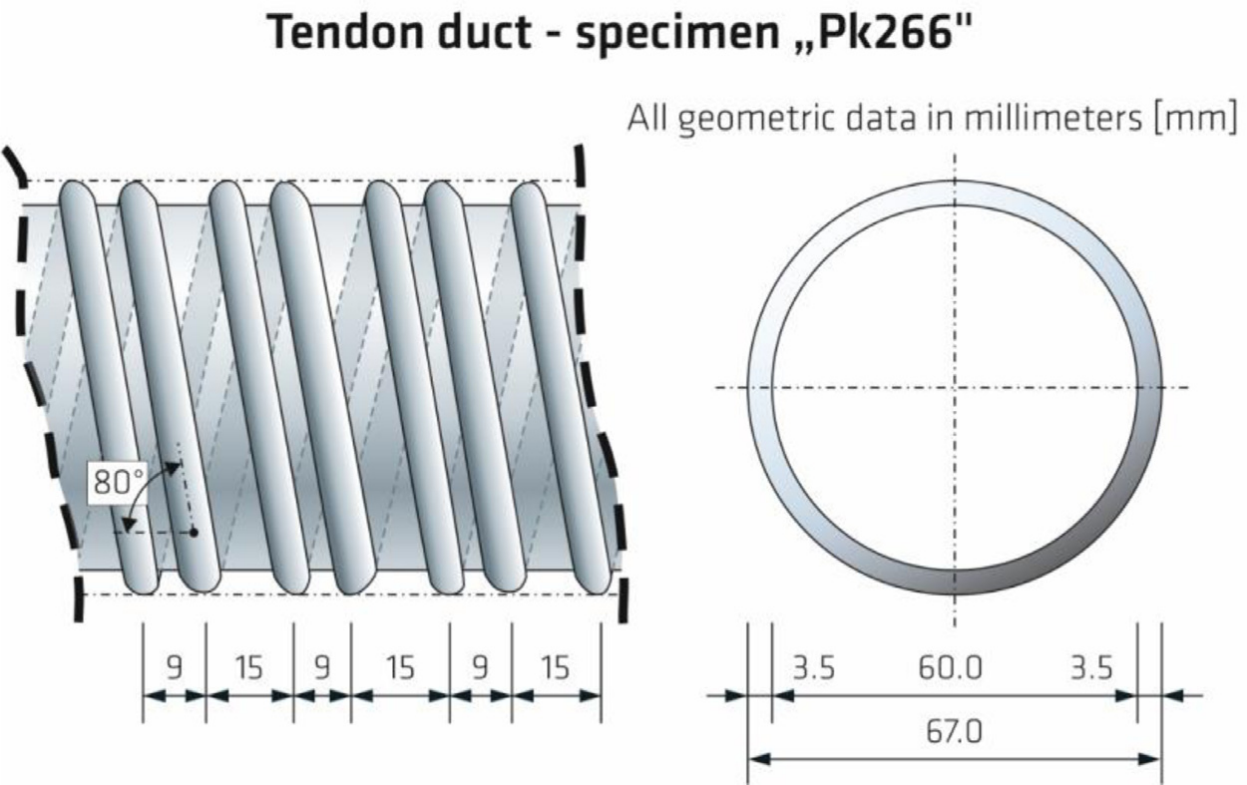
Geometrical dimensions of the tendon ducts installed in test specimen Pk266.

A drawing of the mounted tendon ducts and the associated geometry are shown in Fig. 14.

Table 4 summarizes the geometrical dimensions of test specimen Pk266 based on the chosen coordinate system. The specified thicknesses of the individual component steps include the empirical standard deviation of the thickness measurements performed on twelve measuring points per step. The concrete cover describing the depth position of a tendon duct in Z-direction is the mean value derived from two measurements.

**Table 4:** Geometrical dimensions of the test specimen Pk266 with indication of the standard deviation.

Geometric dimension	X-direction	Y-direction	Z-direction
Length / Width	2000.0 mm	800.0 mm	4 steps
Depth (step 1)	0.0–500.0 mm	800.0 mm	569.9 ± 0.61 mm
Depth (tendon)	250.0 mm	800.0 mm	244.5 ± 0.5 mm
Depth (step 2)	500.0–1000.0 mm	800.0 mm	448.2 ± 1.67 mm
Depth (tendon)	750.0 mm	800.0 mm	184.6 ± 1.6 mm
Depth (step 3)	1000.0–1500.0 mm	800.0 mm	329.8 ± 1.41 mm
Depth (tendon)	1250.0 mm	800.0 mm	121.4 ± 1.0 mm
Depth (step 4)	1500.0–2000.0 mm	800.0 mm	210.3 ± 0.94 mm
Depth (tendon)	1750.0 mm	800.0 mm	64.4 ± 0.75 mm

### 3.5 Ultrasonic Equipment

**Fig. 15:**
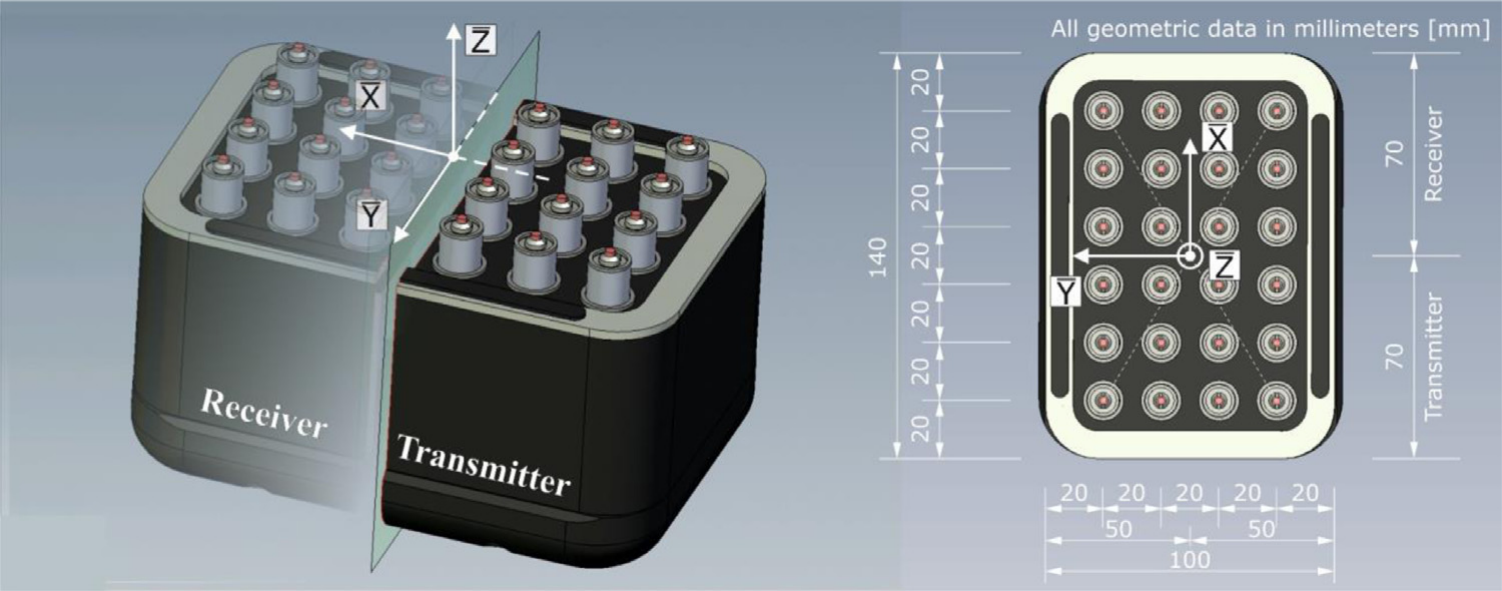
Geometrical dimensions of the probes used for longitudinal wave (M2503) and transversal (SH-shear) wave excitation (M2502); extracted from [8].

The ultrasonic measurements were carried out with probes of the same geometry for both excited wave types. Fig. 15 shows the geometrical probe dimensions and the local coordinate system with its origin in the center of the aperture. A probe consists of 24 spring-mounted individual transducers, 12 of which act as transmitters and the other 12 as receivers. Each of the twelve transducers transmits and receives simultaneously, so that the setup corresponds to a bistatic arrangement (dual-element probe [5]). Each single transducer has a circular contact surface with a diameter of approx. D = 1.7 mm which is placed on the component surface to couple the acoustic pulse into the concrete using the piezoelectric effect.

Fig. 15 shows the geometrical dimensions of the aperture and the transducer spacing. The longitudinal wave excited by the M2503 probe is polarized in Z̅-direction and is excited with a frequency of f = 80 kHz. The transversal (SH-shear) wave excited by the M2502 probe is polarized in Y̅-direction and is excited with a frequency of f = 50 kHz. Both probes have a bandwidth of about 60–70% [9]. The physical values are summarized in Table 5.

**Table 5:** Physical values of probes [9].

Probe type	Wave form	Nom. frequency	Bandwidth	Excited frequency	Polarization
M2503	longitudinal	100 kHz	60–70%	80 kHz	Z̅-direction
M2502	transversal	55 kHz	60–70%	55 kHz	Y̅-direction

**Fig. 16:**
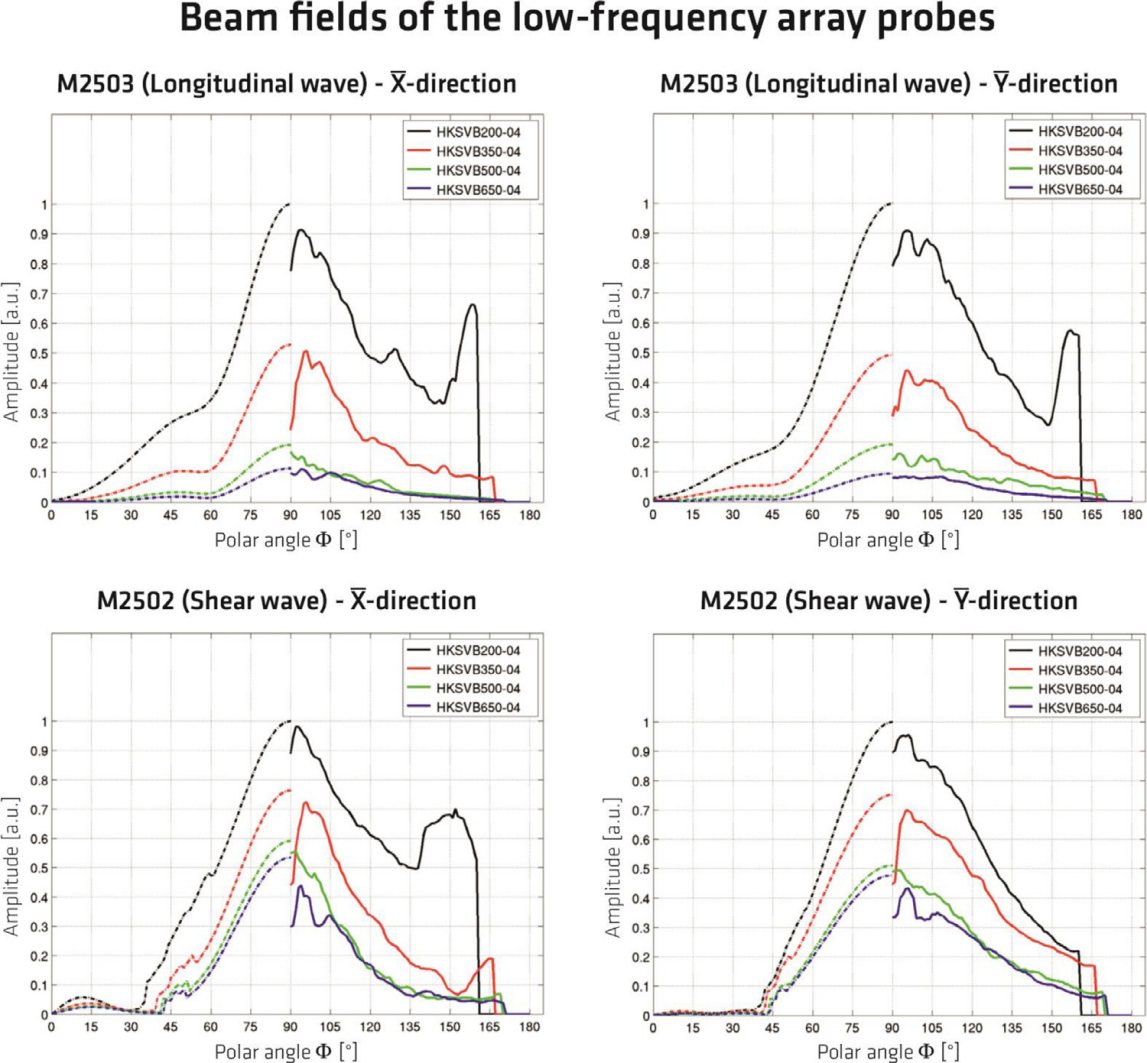
2D visualization of the directivity patterns (solid lines – measurements; dashed lines - simulation) of the applied probes M2502 and M2503 for concrete with a maximum aggregate size of D = 4 mm and different distances form US source. Results extracted from [10]; adapted to the local coordinate system acc. to Fig. 15.

Due to the rectangular arrangement of the individual probes, the excited sound field for both wave types has different characteristics depending on the spatial orientation. The characteristic values of the excited wave types in different planes are visualized in Fig. 16. The diagrams show the results of measurements (solid lines) and simulations (dashed lines) in the respective principal axes. The measurements and simulations were performed at different distances from the surface at which the sound pulse was excited [10]. The difference between the results shown in Fig. 16 and the data collected on the test specimens Pk050 and Pk266 is that the concrete mixture used to describe the sound field characteristics comprises aggregates with a maximum diameter of D = 4 mm [10].

**Fig. 17:**
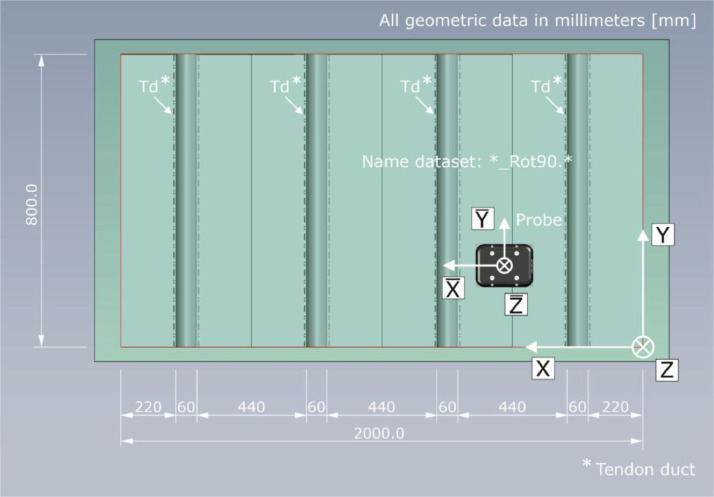
Top view on test specimen Pk266 with probe orientation for the datasets named *_Rot90.*.

**Fig. 18:**
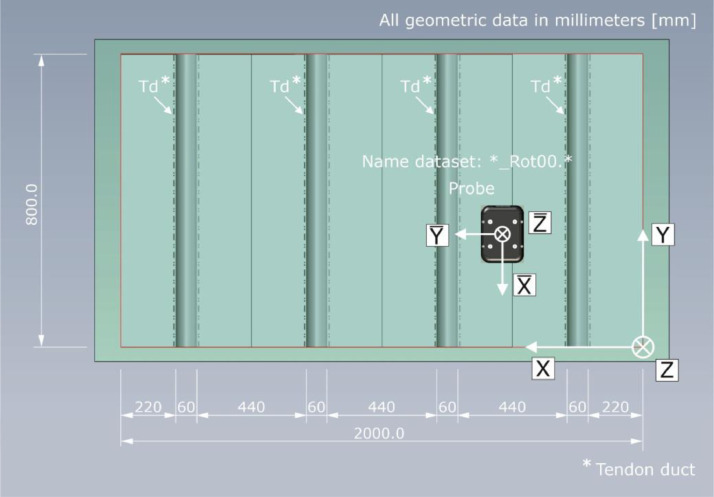
Top view on test specimen Pk266 with probe orientation for the datasets named *_Rot00.*.

Given the spatial dependency of the sound field characteristics shown in Fig. 16, the probe orientation and the resulting designation of the datasets are related to the different coordinate systems in Fig. 17, Fig. 18.

## Ethics Statements

These data are a primary data and does not include any human subjects, animal experiment, or social media platforms.

## CRediT authorship contribution statement

**Stefan Maack:** Conceptualization, Methodology, Investigation, Software, Formal analysis, Writing – original draft, Data curation. **Stefan Küttenbaum:** Formal analysis, Writing – review & editing. **Benjamin Bühling:** Formal analysis, Data curation, Writing – review & editing. **Kerstin Borchardt-Giers:** Visualization. **Norman Aßmann:** Investigation, Writing – review & editing. **Ernst Niederleithinger:** Supervision, Writing – review & editing.

## Declaration of Competing Interest

The authors declare that they have no known competing financial interests or personal relationships that could have appeared to influence the work reported in this paper.

## Data Availability

Low-frequency ultrasound data (pulse-echo technique) with shear horizontal and longitudinal waves on the step-shaped concrete specimen “Pk050” (Original data) (Harvard Dataverse). Low-frequency ultrasound data (pulse-echo technique) with shear horizontal and longitudinal waves on the step-shaped concrete specimen “Pk266” with tendons (Original data) (Harvard Dataverse).
